# Serial crystallography captures enzyme catalysis in copper nitrite reductase at atomic resolution from one crystal

**DOI:** 10.1107/S205225251600823X

**Published:** 2016-06-15

**Authors:** Sam Horrell, Svetlana V. Antonyuk, Robert R. Eady, S. Samar Hasnain, Michael A. Hough, Richard W. Strange

**Affiliations:** a School of Biological Sciences, University of Essex, Wivenhoe Park, Colchester CO4 3SQ, England; b Molecular Biophysics Group, Institute of Integrative Biology, University of Liverpool, Life Sciences Building, Liverpool L69 7ZB, England

**Keywords:** serial crystallography, catalysis, enzyme mechanism, denitrification, copper nitrite reductase, radiation damage, radiolysis, synchrotron radiation, XFEL, MSOX

## Abstract

Relating individual protein crystal structures to an enzyme mechanism remains a major and challenging goal for structural biology. Serial crystallography using multiple crystals has recently been reported in both synchrotron-radiation and X-ray free-electron laser experiments. In this work, serial crystallography was used to obtain multiple structures serially from one crystal (MSOX) to study *in crystallo* enzyme catalysis. Rapid, shutterless X-ray detector technology on a synchrotron MX beamline was exploited to perform low-dose serial crystallography on a single copper nitrite reductase crystal, which survived long enough for 45 consecutive 100 K X-ray structures to be collected at 1.07–1.62 Å resolution, all sampled from the same crystal volume. This serial crystallography approach revealed the gradual conversion of the substrate bound at the catalytic type 2 Cu centre from nitrite to nitric oxide, following reduction of the type 1 Cu electron-transfer centre by X-ray-generated solvated electrons. Significant, well defined structural rearrangements in the active site are evident in the series as the enzyme moves through its catalytic cycle, namely nitrite reduction, which is a vital step in the global denitrification process. It is proposed that such a serial crystallography approach is widely applicable for studying any redox or electron-driven enzyme reactions from a single protein crystal. It can provide a ‘catalytic reaction movie’ highlighting the structural changes that occur during enzyme catalysis. The anticipated developments in the automation of data analysis and modelling are likely to allow seamless and near-real-time analysis of such data on-site at some of the powerful synchrotron crystallographic beamlines.

## Abstract 2

Serial crystallography has been used to drive copper nitrite reductase through its enzymatic cycle while sampling the same volume of a single cryogenically maintained crystal. A structural movie of X-ray-driven enzyme catalysis has thus been obtained, revealing the structural changes that occur during the catalytic reaction in unprecedented detail.

## 1. Introduction   

A major objective in structural biology is to determine accurate structures of all relevant intermediates in an enzyme mechanism. This ambition has only been partially fulfilled in most cases owing to difficulties in obtaining crystals in the relevant reaction states or to differences between individual crystals, as well as the deleterious effects of X-ray radiation damage/radiolysis, which has long been a concern in structural biology (Ravelli & Garman, 2006 ▸; Garman, 2010 ▸; Garman & Weik, 2015 ▸). However, only relatively recently has it become widely recognized that the reduction of redox centres by X-ray-generated photoelectrons can occur very rapidly, often prior to significant loss of diffraction resolution, damage to disulfide bridges, thiols or amino-acid side chains (Beitlich *et al.*, 2007 ▸; Antonyuk & Hough, 2011 ▸; Yano *et al.*, 2005 ▸). Such X-ray-induced changes to the redox states of metal centres can lead to mis-assignment of functional states or steps in a catalytic mechanism to a particular crystal structure. In some cases, this problem has been turned into an opportunity by limiting the absorbed X-ray dose and using X-ray radiolysis to drive the reaction and obtaining structures in specific redox states which may be relevant to catalytic or other protein mechanisms (Schlichting *et al.*, 2000 ▸; Hough *et al.*, 2008 ▸). This approach has more recently been used to characterize X-ray-generated species in, for example, tobacco assimilatory nitrite reductase (Nakano *et al.*, 2012 ▸; an Fe–S cluster- and sirohaem-containing protein that converts nitrite to ammonium), horseradish peroxidase and multicopper oxidases (Berglund *et al.*, 2002 ▸; Komori *et al.*, 2014 ▸; De la Mora *et al.*, 2012 ▸).

Serial crystallography performed at X-ray free-electron lasers (XFELs) using multiple protein crystals has recently seen major advances in this direction (Nogly *et al.*, 2015 ▸; Suga *et al.*, 2015 ▸; Schlichting, 2015 ▸; Kern *et al.*, 2014 ▸), with some of the same methodology also being proposed for multiple-microcrystal synchrotron-radiation experiments (Gati *et al.*, 2014 ▸; Stellato *et al.*, 2014 ▸). The recent introduction of the fast, shutterless Pilatus series of pixel-counting area X-ray detectors (Rajendran *et al.*, 2011 ▸) has provided the capability for rapid measurement. Using this capability, we have collected a large number (many tens) of serial crystallographic data sets at near-atomic resolution from the same region of a *single* protein crystal in a reasonable amount of experimental time. This represents a ‘multiple structures serially obtained from one crystal’ (MSOX) approach rather than a ‘serial crystals–one structure’ (SFX) approach pioneered using femtosecond XFEL sources. We exploit this methodology to allow the production of a many-framed, dose-dependent series of structures from a single protein crystal. This has enabled us to obtain a ‘structural movie’ of catalysis *via* the progressive addition of X-ray-generated solvated electrons to the crystalline protein. Importantly, the occurrence of changes to X-ray-induced redox states occurs at much lower X-ray doses than ‘classical’ radiation damage and this enables a single crystal to ‘survive’ long enough to allow many structures of relevant states to be determined while the crystal continues to diffract to high resolution. A major advantage of MSOX is that transitions of structural changes are captured like a ‘real movie’ as the radiolysis drives the enzyme from one state to the next in a smooth manner, rather than capturing snapshots where subtle changes may not be considered to be significant or may be missed altogether.

Copper nitrite reductase (CuNiR) is part of the microbial respiratory N-oxide reduction denitrification pathway and catalyses the reduction of nitrite to form nitric oxide, a process with key agronomic and environmental impacts (Zumft, 1997 ▸; Eady & Hasnain, 2003 ▸; Maia & Moura, 2014 ▸). For example, the NO generated by CuNiR is converted by nitric oxide reductases (NOR) into the potent greenhouse gas N_2_O, which is increasingly being recognized as a major player in climate change. CuNiRs are homotrimers, where each monomer contains a type 1 Cu centre (T1Cu) with a role in electron transfer and a catalytic type 2 copper centre (T2Cu) where nitrite binds and is converted to nitric oxide. These centres are ∼12.6 Å apart and in the absence of nitrite are characterized by a low driving force for electron transfer. The T2Cu centre has Cu(His)_3_–H_2_O ligation and nitrite binding displaces the H_2_O ligand. Mechanistic studies on a number of CuNiRs have given seemingly contradictory data as to whether intramolecular electron transfer occurrs before or after nitrite binding. These data have largely been reconciled in a kinetic scheme for catalytic turnover in which two parallel routes differing in the sequence of electron transfer and binding of nitrite are possible, depending on the pH and nitrite concentration (see Krzemiński *et al.*, 2011 ▸). A combined kinetic, EPR and EXAFS study of ligand binding in *Alcaligenes xylosoxidans* CuNiR (*Ax*NiR) provided the first clear evidence that the enzymatic reaction proceeds *via* an ordered mechanism rather than a random mechanism. In addition, it was shown that nitrite binds to the oxidized T2Cu centre before electron transfer from the reduced T1Cu centre occurs, otherwise the enzyme ends up as a dead-end species (Strange *et al.*, 1999 ▸). The presence of this ‘trigger’ mechanism for electron transfer from T1Cu to the catalytic site in *Ax*NiR initiated by the binding of substrate to the catalytic site has been confirmed by combined crystallographic and online spectroscopy (Hough *et al.*, 2008 ▸) and by laser flash photolysis rapid kinetic experiments (Leferink *et al.*, 2011 ▸, 2012 ▸).

We have previously shown that in the absence of nitrite the T2Cu centre in crystals of *Ax*NiR remains in the four-coordinate oxidized, copper(II) state following an X-ray dose that was significantly in excess of that required for complete reduction of T1Cu. In contrast, partial turnover of nitrite to NO at the T2Cu centre occurred when substrate was present (Hough *et al.*, 2008 ▸). This result raised the intriguing possibility that X-ray radiolysis may be used to capture catalytic turnover occurring in crystals, allowing the catalytic mechanism to be followed *via* a series of high-resolution crystal structures. The generation of solvated electrons within a cryocooled crystal as a result of X-ray irradiation (Berglund *et al.*, 2002 ▸) acts as a surrogate for the electrons that would be provided *in vivo* by a physiological electron-donor protein (in *Ac*NiR this is the cupredoxin pseudoazurin). We have measured a series of 45 consecutive serial data sets using an *Ac*NiR crystal with, initially, nitrite bound to the T2Cu site, allowing us to follow X-ray-driven catalysis in the crystals and to define reaction intermediates through the catalytic cycle. Each of the structures was obtained with a total exposure time of 19 s and an average dose of 0.69 MGy using a 90 × 45 µm X-ray beam. Our serial structures show the promise of MSOX crystallo­graphy for studying structure-based enzyme mechanisms in redox systems.

## 2. Methods   

### 2.1. Protein production and purification   

A pET-26b plasmid containing the *Ac*NiR gene with codon optimization for expression in *Escherichia coli* was purchased from GenScript Inc. Overexpression was performed in *E. coli* BL21 (DE3) cells as described previously (Chen *et al.*, 1996 ▸). Cells were lysed by sonication and centrifuged at 9400*g* for 15 min at 4°C. Cleared lysate was dialysed against 2 m*M* CuSO_4_ in 20 m*M* Tris–HCl pH 7.5 at 4°C followed by a second round of dialysis against 20 m*M* Tris–HCl pH 7.5 at 4°C, giving the resulting lysate a distinct green colour. This was loaded onto a DEAE cellulose column equilibrated with 20 m*M* Tris–HCl pH 7.5 and washed with 10 m*M* potassium phosphate. *Ac*NiR was eluted with a linear gradient of potassium phosphate from 10 to 150 m*M*, with the green *Ac*NiR fraction followed visually. *Ac*NiR was further purified by ammonium sulfate precipitation by gradually increasing the ammonium sulfate concentration to approximately 1.4 *M*. Protein purity was assessed by SDS–PAGE. The protein solution was buffer-exchanged into 50 m*M* MES buffer pH 6.5 prior to crystallization.

### 2.2. Crystallization and nitrite soaking   

Crystals of recombinant *Ac*NiR in space group *P*2_1_3 were grown to approximately 300 × 200 × 200 µm in size, as described previously for wild-type *Ac*NiR (Antonyuk *et al.*, 2005 ▸). Crystals were soaked in a solution consisting of 0.1 *M* sodium nitrite, 2.4 *M* ammonium sulfate, 0.1 *M* sodium acetate pH 5.5 for 1 h at room temperature before being transferred into a cryoprotectant solution consisting of 0.1 *M* sodium nitrite, 3.5 *M* sodium malonate pH 5.0 and cryocooled by plunging into liquid nitrogen.

### 2.3. Serial crystallographic data collection   

The rapidity of the serial data-collection method allowed data to be measured from several nitrite-soaked *Ac*NiR single crystals in succession, and for each crystal to be evaluated for the presence of the nitrite ligand bound to the type 2 Cu site. We report in detail one such series of 45 consecutive 100 K data sets that were measured from *one* of these nitrite-soaked crystals on beamline I04 at Diamond Light Source with an X-ray wavelength of 0.97 Å using a Pilatus 6M-F (Dectris) detector (Kraft *et al.*, 2009 ▸). Each data set in the series (denoted ds1 to ds45) was collected using the same oscillation range and crystal position in order to ensure that the same crystal volume was irradiated. A total angular range of 38° was collected for each data set, with 0.1° oscillation and 50 ms exposure per image and with a 90 × 45 µm beam, to give a total exposure time of 19 s and an average dose of 0.69 MGy per data set as calculated using *RADDOSE*-3*D* (Zeldin *et al.*, 2013 ▸) based on a measured flux of 2 × 10^12^ photons s^−1^. The shutterless data-collection mode and small oscillation per image resulted in only partial reflections being recorded.

### 2.4. Data processing and refinement   

The initial data sets were processed within the automated pipeline at Diamond Light Source using *xia*2 (Winter *et al.*, 2013 ▸) to rapidly assess the presence of bound nitrite. All 45 data sets from the crystal were then reprocessed using *XDS* (Kabsch, 2010 ▸) and *AIMLESS* (Evans & Murshudov, 2013 ▸) in the *CCP*4 suite (Winn *et al.*, 2011 ▸). The first data set, ds1, was obtained to 1.07 Å resolution based on the CC_1/2_ > 0.5 cutoff (Karplus & Diederichs, 2012 ▸) and 〈*I*/σ(*I*)〉 > 1 in the outermost resolution shell. Merging statistics for selected data sets from the series are presented in Table 1 ▸.

The structure associated with the first data set, ds1, was refined in *REFMAC*5 (Murshudov *et al.*, 2011 ▸) using the 1.10 Å resolution structure of wild-type *Ac*NiR as a starting model (PDB entry 2bwi; Antonyuk *et al.*, 2005 ▸) but with the ligands removed. 5% of the data were excluded from refinement to calculate the free *R* factor (Brünger, 1992 ▸). Models were rebuilt interactively and water and ligand molecules were added into σ_A_-weighted 2*F*_o_ − *F*_c_ and *F*_o_ − *F*_c_ electron-density maps using *Coot* (Emsley *et al.*, 2010 ▸). No restraints were applied to the Cu–ligand distances or bond angles. Occupancies of amino-acid side chains and ligand/water molecules at the T2Cu site were assessed using electron-density OMIT maps and analysis of their *B* factors relative to those of surrounding atoms. Individual anisotropic temperature factors were refined for structures with a resolution limit of 1.4 Å or higher. The electron-density map was modelled with two main-chain conformations in the region between Lys195 and Ala202.

This refined and validated structure, with ligands again removed, was used as the starting model for refinement of the second data set, ds2. Subsequently, the final model from refinement of each data set was used as the starting model for the next data set in the series as the resolution decreased. Stereochemical quality and fits to the electron density of the final models for each of the 45 data sets were assessed at each step of this refinement process using *MolProbity* (Chen *et al.*, 2010 ▸), *JCSG Quality Control Check* and *Coot* (Emsley *et al.*, 2010 ▸). Figures were rendered using *PyMOL* (v.1.8; Schrödinger).

## 3. Results and discussion   

### 3.1. Serial data-collection statistics   

Increasing absorbed dose delivered by the measurement of serial data sets from the same region of the nitrite-soaked crystal caused a steady reduction in the high-resolution limit and an increase in overall *B* factors and unit-cell parameters consistent with previous radiation-damage studies. The high-resolution limit decreased from an atomic resolution of 1.07 Å to 1.45 Å in ds30 upon exceeding the Henderson limit of 20 MGy (Henderson, 1990 ▸). All data collection was completed prior to reaching the Garman limit of 43 MGy (Owen *et al.*, 2006 ▸). The conversion of nitrite to NO occurred well before the Henderson dose limit was reached and with barely any loss of resolution. As expected, overall mean *B* factors for all protein atoms showed a steady increase in line with increasing average diffraction weighted dose from 13 to 31 Å^2^ over the course of the series. The atomic resolution of ds1 allowed two positions of nitrite to be modelled with a high level of certainty; however, modelling became more challenging beyond the Henderson limit (ds30) as the quality of the electron-density maps deteriorated.

### 3.2. Structures of *Ac*NiR and ligand modelling   

The overall *Ac*NiR structure determined from ds1 showed no significant differences (all-atom r.m.s.d. of 0.14 Å) when compared with previously published atomic resolution nitrite-bound structures (Antonyuk *et al.*, 2005 ▸), and the tertiary structure remained essentially unchanged throughout the data-collection series, with an r.m.s.d. of between 0.09 and 0.12 Å for all atoms when compared with ds1. The T1Cu site showed only limited structural changes throughout the dose series (Supplementary Table S1), consistent with the entatic state requiring only a very small reorganization energy between the copper(II) and copper(I) states (Williams, 1995 ▸). The most prominent structural change of the protein at the T2Cu site (Table 2 ▸) involved movement of Asp98 between the proximal and gatekeeper alternate conformations, as first observed in atomic resolution structures of *Ac*NiR by Antonyuk *et al.* (2005 ▸). In these structures water-bound *Ac*NiR (PDB entry 2bw5) and nitrite-bound *Ac*NiR (PDB entry 2bwi) both showed dual conformations of Asp98, while endogenous nitric oxide-bound *Ac*NiR (PDB entry 2bw4) displayed the proximal conformation only (Antonyuk *et al.*, 2005 ▸).

**Figure 1:**
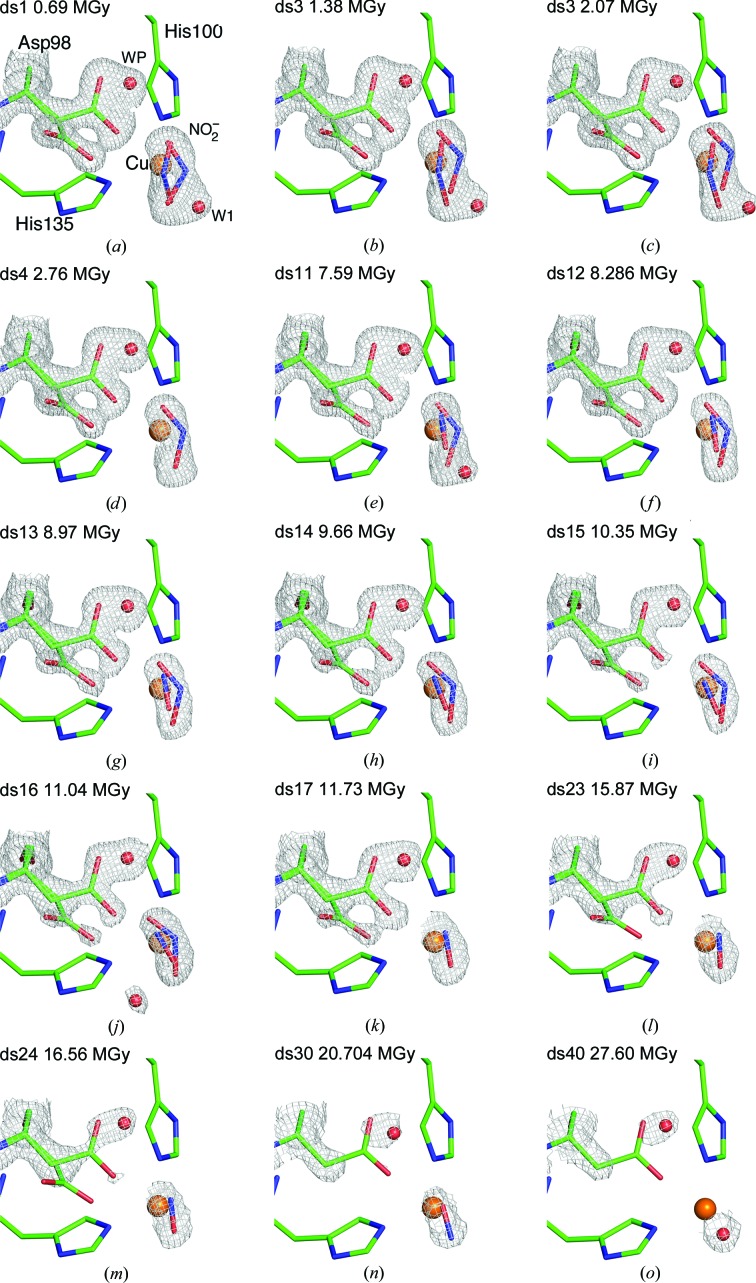
Movie frames for X-ray-induced ligand turnover in *Ac*NiR, showing the conversion of nitrite to nitric oxide. The proximal and gatekeeper positions of Asp98 are modelled in the electron-density maps. (*a*) ds1 with dual-occupancy nitrite and a water molecule, (*b*, *c*) ds2, ds3 with dual-occupancy nitrite, (*d*) ds4 with single-occupancy nitrite in the side-on conformation, (*e*) ds11 with dual-occupancy nitrite and nitric oxide with water, (*f*–*i*) ds12–ds15 with dual-occupancy nitrite and nitric oxide, (*j*) ds16 with dual-occupancy nitrite and nitric oxide plus water, (*k*, *l*, *m*) ds17–ds24 with nitric oxide, (*n*) ds30 with nitric oxide and single-conformation Asp98 in the proximal position, (*o*) ds40 with bound water. Asp98, His100, His135, nitrite and nitric oxide are represented as sticks and water molecules and Cu atoms as spheres. 2*F*_o_ − *F*_c_ density is contoured in the range 0.53–0.36 e Å^−3^.

Two nitrite molecules at 40% occupancy each (Fig. 1 ▸; see also Supplementary Fig. S1 for difference density maps), with similar *B* factors for each of the constituent atoms, were modelled at the T2Cu site for ds1. By ds4 (2.76 MGy accumulated dose) the nitrite had stabilized into a single ‘side-on’ conformation as observed previously in wild-type *Ac*NiR structures (Antonyuk *et al.*, 2005 ▸). The observation of a new ‘top-hat’ conformation of nitrite at 1.07 Å resolution in ds1 was enabled by the low-dose (0.69 MGy) synchrotron-radiation data collection using a low-noise detector. Data set ds1 has the least radiation damage of all data sets in the series and the top-hat conformation here matches the vertical substrate coordination observed in *Alcaligenes faecalis* NiR in a recent report using serial femtosecond ‘radiation-damage-free’ crystallography at the SACLA XFEL (Fukuda *et al.*, 2016 ▸). In subsequent data sets, as the enzyme reaction advanced using the photoelectrons generated by the X-rays and NO was produced (ds11), there was only a minor loss of resolution owing to radiation damage (the data resolution remained within 0.02 Å of that of the starting structure). Although heavy metal sites such as Cu can provide hotspots for local radiation damage this does not appear to be the case for CuNiRs since the T1Cu site showed only limited structural changes throughout the dose series (Table S1). In the case of the catalytic site many high-dose synchrotron X-ray structural studies have shown the T2Cu centre to have Cu-(His)_3_–H_2_O ligation in accord with ^14^N and ^1^H ENDOR solution studies, (Howes *et al.*, 1994 ▸; Veselov *et al.*, 1998 ▸). This indicates that the first coordination sphere ligation is not changed by exposure to X-rays and that the changes we observe are due to catalysis at the T2Cu site following transfer of an electron from the radiolytically reduced T1Cu site.

The top-hat coordination state must represent the initial position of the bound nitrite. This position of nitrite is still accompanied by the gatekeeper conformation of Asp98 that is responsible for delivery of nitrite to the catalytic pocket. With increased radiolysis, in ds4 at 1.09 Å resolution the top-hat conformation is converted to a single side-on nitrite conformation, observed as before with Asp98 mostly in its proximal position, ready to participate in O—N—O bond breakage (Fig. 1 ▸ *d*). Since no product is formed during this conversion, the shift of Asp98 to its proximal position presumably reflects changes in the oxidation state of the Cu centres or a protonation event. Whatever the nature of the triggering event, these data provide the first direct experimental evidence for the operation of the sensor loop (Hough *et al.*, 2005 ▸) during catalysis.

**Figure 2:**
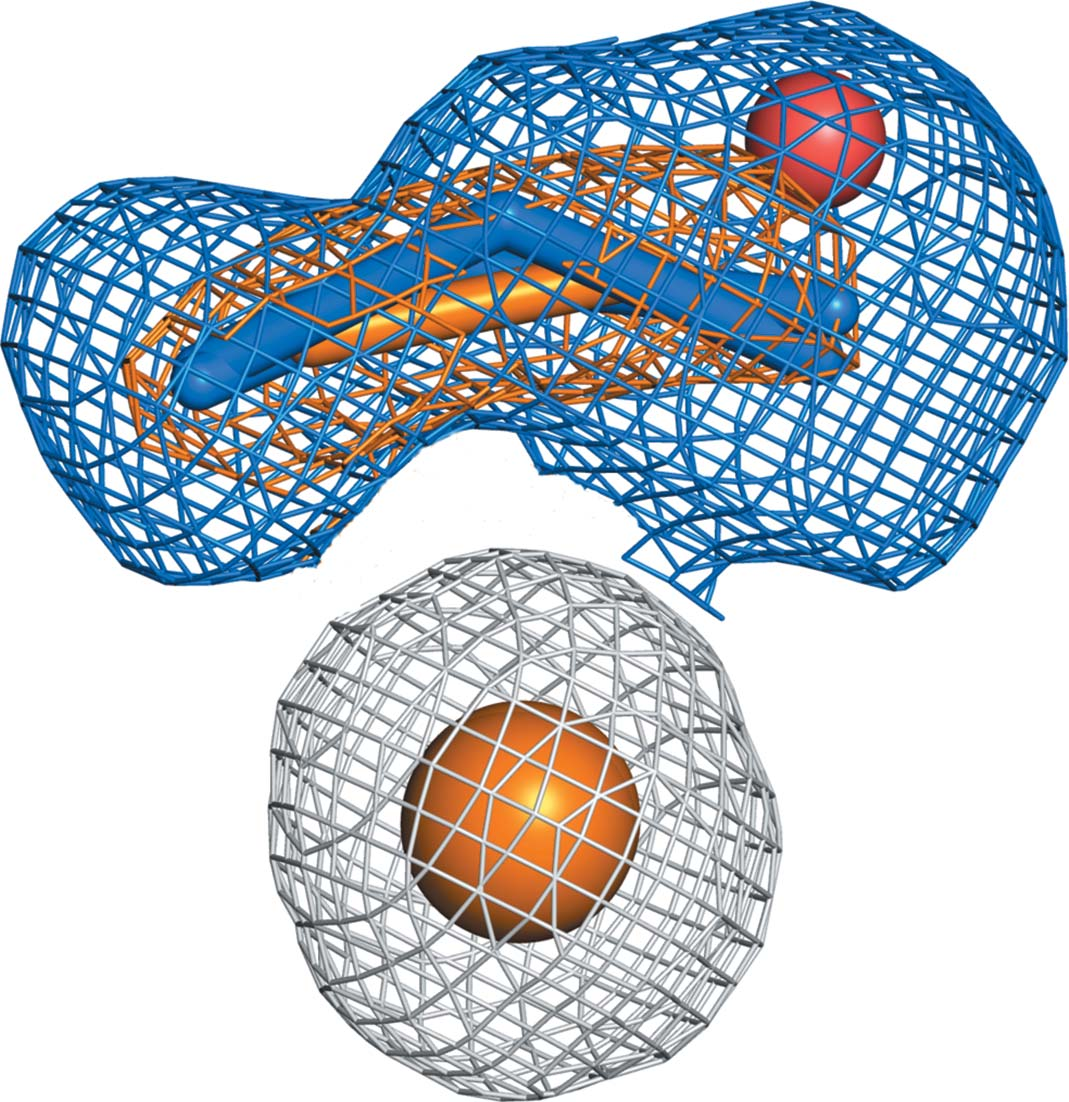
The T2Cu site of ds1 with ‘top-hat’ and ‘side-on’ nitrite orientations (blue sticks) modelled in the 2*F*_o_ − *F*_c_ electron-density map (blue mesh) at 1.07 Å resolution, superimposed against the electron density corresponding to a ‘side-on’ nitric oxide product in ds17 (orange sticks and mesh) at 1.22 Å resolution. Both maps are contoured at 1σ.

From ds11 (7.59 MGy), with the crystal still diffracting to 1.09 Å resolution, a new positive difference electron-density feature was observed at the T2Cu site. Based on the shape of the density and the dual conformation of Asp98, which is not characteristic of either fully occupied nitrite or nitric oxide, the active site was modelled with partial occupancies of NO_2_/NO and water. From ds17 a single nitric oxide molecule was present in the structure (Fig. 1 ▸ *k*). Fig. 2 ▸ summarizes these ligand-bound models and shows the difference in the electron density at the T2Cu site between ds1 and ds17. The resulting sequential structures were used to generate a catalytic movie (Supplementary Movie S1). This structural movie demonstrates several key steps of catalysis unambiguously from the initial ‘top-hat’ and ‘side-on’ nitrite-binding modes, to product formation, loss of nitrite and product release.

### 3.3. Structural rearrangements at the catalytic site during enzyme catalysis   

Over the course of the X-ray dose series of 45 serial structures, the T2Cu catalytic centre passes through several structural changes that we were able to observe as a result of the high to atomic resolution of the structures in a single pass and the short dose increments between data sets enabled by the faster detector system. Over the first three data sets nitrite appears to occupy two alternate positions, both with bidentate oxygen binding to Cu (Table 2 ▸) and related by a rotation horizontally, with the O1 and O2 atoms almost invariant and the N atom moving between ‘top-hat’ NO_2_a and ‘side-on’ NO_2_b positions in Figs. 1 ▸(*a*)–1 ▸(*c*). These alternate orientations provide a rationale for the ∼60% higher *B* factors observed for the central N atom of nitrite relative to the O atoms when modelled as a single high-occupancy nitrite molecule. By ds4, the nitrite population converges into the ‘side-on’ NO_2_ conformation similar to that observed in previous studies (Antonyuk *et al.*, 2005 ▸), where it remains for a further six data sets. The conformation of Asp98 favours the proximal position at ∼0.6 occupancy between ds4 and ds10 (Supplementary Fig. S2). At the end of ds10 the exposed volume of the crystal had received an accumulated dose of 6.9 MGy, which is 30% of the Henderson limit, and still gave diffraction to ∼1.1 Å resolution.

In ds11, a previously unseen T2Cu ligand conformation was observed, with a new water molecule (W2) elongating the electron-density feature above the T2Cu. In this structure an intermediate reaction stage with nitrite, nitric oxide and water was modelled (Fig. 1 ▸ *e*). This model is based on resurgence of the electron density for the gatekeeper conformation of Asp98 to 50% occupancy, whereas the proximal position had been more prevalent in preceding data sets. A similar distribution of dual conformations of Asp98 was observed with a mixture of nitrite and nitric oxide ligands in as-isolated *Ac*NiR (Antonyuk *et al.*, 2005 ▸; PDB entry 2bwd). The resurgence of the gatekeeper conformation may indicate its dual role in substrate delivery and product release.

In the next six serial data sets (Figs. 1 ▸ *e*–1 ▸ *j*) nitrite and nitric oxide were modelled with the same occupancies and positions, with a water molecule also present close to the ligand-binding site in ds11 and ds16. The intermittent appearance of this water suggests that it may represent the final link in the water chain that allows proton transfer to T2Cu and facilitates the second protonation and cleavage of the nitrite and formation of product NO. By ds16 the crystal had been exposed to 11.04 MGy, which is 55% of the Henderson limit, and the high-resolution limit was 1.15 Å.

From ds17 onwards a single nitric oxide molecule was observed at the T2Cu site (Fig. 1 ▸ *k*) and the conformation of Asp98 favoured the proximal position, with 60–70% occupancy up to ds30, where the gatekeeper conformation was no longer observed. The 2*F*_o_ − *F*_c_ density at 1σ typical of bound nitrite (Supplementary Fig. S3) has become flatter relative to the Cu atom. By ds17 the crystal had been exposed to 11.7 MGy and the resolution limit was 1.22 Å.

The mode of binding of the enzymatically generated NO product to CuNiRs has attracted much attention. In contrast to the end-on mode of binding predicted by spectroscopic and computational studies (Periyasamy *et al.*, 2007 ▸; Sundararajan *et al.*, 2007 ▸; Silaghi-Dumitrescu, 2006 ▸; De Marothy *et al.*, 2007 ▸; Li *et al.*, 2015 ▸), a side-on mode was proposed based on the structure of NO-soaked CuNiR from *A. facaelis* (Tocheva *et al.*, 2004 ▸). Even though the side-on NO-binding mode received support from an atomic resolution *Ac*NiR structure with endogenous bound NO (Antonyuk *et al.*, 2005 ▸), the significance of this finding has continued to be questioned in the computational chemical community. The study of Tocheva and coworkers was further complicated by the non-enzymatic origin of the ligand and the discovery that the nitrite reductase reaction is reversible, with exposure to excess NO gas producing a T1Cu(red)–T2Cu nitrite species (Ghosh *et al.*, 2007 ▸). It has been suggested that the side-on NO coordination seen in crystals represents a metastable form that relaxes to end-on coordination in solution when the positions of the side chains of the active-site pocket residues Asp_CAT_ and isoleucine are more flexible (Solomon *et al.*, 2014 ▸). The structures presented here provide powerful evidence for the AspCAT residue in not modulating the formation of the side-on NO product molecule, observed with near equidistant Cu—N and Cu—O bonds, during the conversion of substrate nitrite.

### 3.4. The role of Asp98 in ligand sensing   

The close correlation of the occupancies of the proximal conformation of Asp98 and the coordinated nitrite molecule indicates that Asp98 plays a central role in catalysis other than simply acting as a provider of a proton. Over the course of the first 30 data sets the occupancy of the proximal conformation of Asp98 first increases as the population of nitrite decreases, leaving the nitrite molecule only in the side-on conformation (ds4–ds10; substrate delivery and anchoring), decreases in the presence of dual-conformation nitrite and NO (ds11–ds16 product formation) and then rises again as the nitrite is lost, leaving a T2Cu–NO occupied active site (ds17–ds30) (Figs. 1 ▸ *a*–1 ▸ *m*). These data suggest that Asp98 plays a role in sensing, guiding and stabilizing the T2Cu substrate within the catalytic pocket, providing the proton in the product (NO) formation step and perhaps is involved in its release with concomitant return of the enzyme to the resting state.

**Figure 3:**
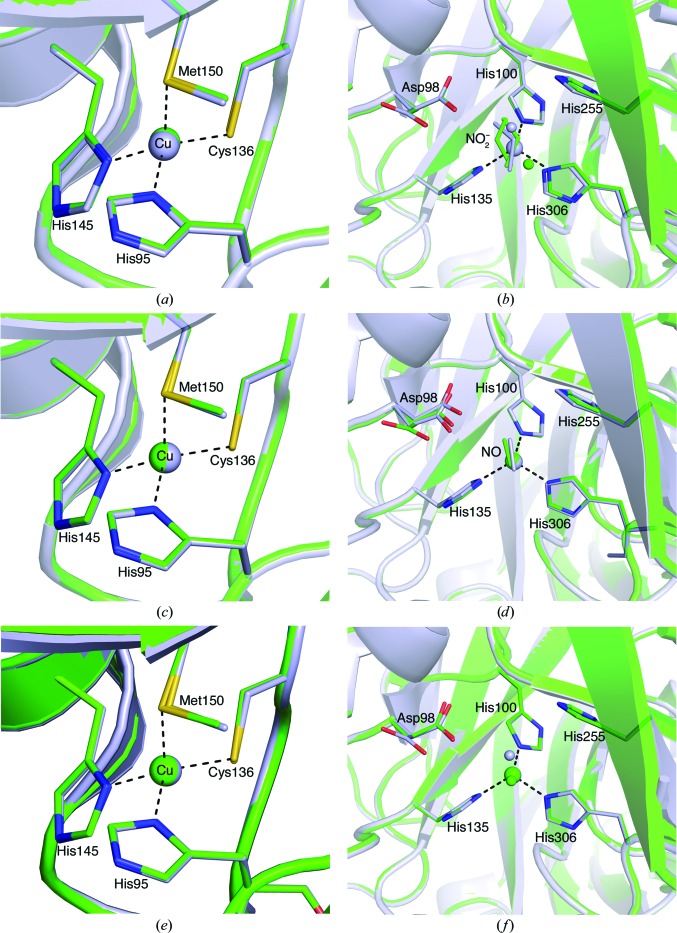
Superpositions of the T1Cu and T2Cu sites of serial data-collection models (green) against published nitrite-bound, nitric oxide-bound and native *Ac*NiR structures (grey): ds1 and PDB entry 2bwi for (*a*) the T1Cu site and (*b*) the nitrite-bound T2Cu site; ds17 and 2bw5 for (*c*) the T1Cu site and (*d*) the nitric oxide-bound T2Cu site; ds40 and 2bw4 for (*e*) the T1Cu site and (*f*) the T2Cu site.

Comparison of serial structures with corresponding individual structures from previous studies show similar, although not identical, positioning of Asp98 with T2Cu ligands (Antonyuk *et al.*, 2005 ▸). The T1Cu and T2Cu atoms and liganding residues are almost identical in all cases (Figs. 3 ▸ *a*–3 ▸ *f*). Endogenous nitrite-bound *Ac*NiR (PDB entry 2bwi) and nitrite-soaked *Ac*NiR from serial data collection both show dual conformations of Asp98. Dual conformations are likely to stem from the incomplete incorporation of nitrite in both crystals (80% in ds1 and 50% in 2bwi), as water-bound *Ac*NiR also exhibits a dual-conformation Asp98 (PDB entry 2bw4). Endogenous nitric oxide-bound *Ac*NiR (PDB entry 2bw5) shows two slightly different conformations of Asp98 in the proximal position, while ds17 shows both the proximal and gatekeeper dual conformation of Asp98, although the occupancy does favour the proximal position. Comparison of the final stages of the reaction, where the product has left and has been replaced by a water molecule, is more difficult to ascertain with certainty owing to the loss of resolution resulting from the high dosage of the crystal by ds40 (138% of the Henderson limit). ds40 shows only the proximal conformation with a reduced occupancy (0.55). Whether this is a significant conformational change is unclear, as a loss of density in ds40 was also observed in four other Asp residues distant from the T2Cu centre (Asp8, Asp89, Asp160 and Asp230), while the 15 other Asp residues in each subunit of the trimer showed no discernible loss of electron density.

### 3.5. Insights into the mechanism of nitrite reduction from serial structures   

The catalytic mechanism of CuNiRs from several bacterial organisms has been studied crystallographically (Boulanger & Murphy, 2001 ▸; Tocheva *et al.*, 2004 ▸, 2007 ▸; Antonyuk *et al.*, 2005 ▸; Jacobson *et al.*, 2007 ▸), spectroscopically (Goldsmith *et al.* 2011 ▸; Krzemiński *et al.*, 2011 ▸; Solomon *et al.*, 2014 ▸) and by kinetic and mutation studies (Wijma *et al.*, 2006 ▸; Hough *et al.*, 2008 ▸; Leferink *et al.*, 2014 ▸). Through these studies it has been shown that the reaction consumes one electron, which is transferred from reduced T1Cu to T2Cu *via* the Cys135–His136 bridge, and two protons thought to be provided directly by Asp98 and indirectly by His255 through a bridging water molecule. Based on high-resolution crystal structures of water-bound, nitrite-bound and nitric oxide-bound CuNiRs, a reaction mechanism has been proposed (Antonyuk *et al.*, 2005 ▸) in which nitrite binds to the T2Cu in a bidentate conformation *via* equidistant Cu—O bonds, displacing the native bound water and facilitating electron transfer from T1Cu to T2Cu. Bound nitrite is then cleaved through protonation to give nitric oxide and water. Nitric oxide leaves the active site and water binds to T2Cu to regenerate the resting-state enzyme.

X-ray-induced catalysis using serial data collection from a single nitrite-bound *Ac*NiR crystal has revealed several intermediate binding conformations of nitrite as it proceeds through the reaction. Initially nitrite binds to the T2Cu in two conformations, appearing to pivot between a vertical ‘top-hat’ conformation, with bidentate oxygen binding and the central N pointing upwards away from the Cu atom, and the side-on conformation observed previously (Antonyuk *et al.*, 2005 ▸), before settling into the side-on conformation. Computational simulations of the reaction mechanism suggest a monodentate N-bound nitrite molecule following reduction of T2Cu (Li *et al.*, 2015 ▸); however, no such conformation was observed over this series of structures and instead the side-on nitrite is converted into the side-on nitric oxide product. If an N-coordinated intermediate is present our data imply that it must be very transient in nature and has escaped the time-scales of serial crystallography recorded here. Our study also reveals a previously unseen dual conformation of nitrite and nitric oxide with an extended water network (‘proton tube’) linking the T1Cu and T2Cu sites with bulk solvent (see Fig. 5) that may represent an alternative mechanism by which the protonation of nitrite occurs.

### 3.6. Communication between T1Cu and T2Cu   

Analysis of the behaviour of the proposed ‘sensor loop’ residues 95–100 (Hough *et al.*, 2005 ▸) for the 45 structures showed that there was no significant structural change while nitrite remains bound. Asp98 exhibits the only dual conformation within the sensor loop, and as described above the balance between the gatekeeper and proximal positions changes in the early stages of the reaction. From ds11 onwards, with dual-occupancy nitrite and nitric oxide, deviations in the peptide backbone between Ile97 and Asp98 emerge, with two distinct backbone conformations 0.2 Å apart. This backbone conformation is still present in ds17, where all of the nitrite has been converted to nitric oxide. This subtle backbone movement may be indicative of conformational changes being signalled between the Cu sites during catalysis. Such positional effects may be constrained at the cryogenic temperatures used in the experiments; for example, suppression of side-chain flipping following metal redox-state changes at 100 K has been observed previously (Kekilli *et al.*, 2014 ▸). Temperature-controlled kinetic crystallography may be used to trap proteins in functionally relevant intermediate stages in a reaction using trigger–cool or cool–trigger techniques (Ringe & Petsko, 2003 ▸; Weik & Colletier, 2010 ▸), such as in acetyl­choline esterase, where structural changes are observed at 155 K but not at 100 K (Weik *et al.*, 2001 ▸).

**Figure 4:**
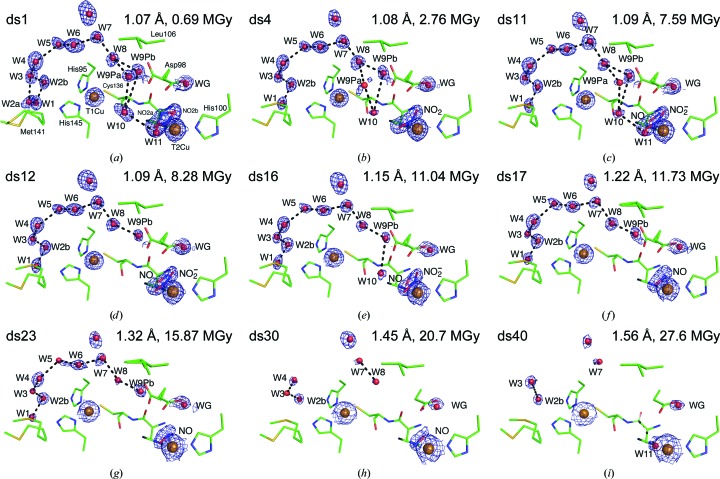
Structural movie of the changes observed in the water chain extending from the T1Cu to T2Cu sites of serial data sets ds1–ds40. All 2*F*_o_ − *F*_c_ density is contoured at 1σ. Dashed lines represent hydrogen bonds forming putative proton-transport pathways between water molecules. The waters labelled W9P and WG represent the partial-occupancy waters that are present when the proximal and gatekeeper conformations of Asp98, respectively, are observed. All residues, nitrite and nitric oxide are represented as sticks and water and Cu atoms are represented as as spheres; all are coloured by element.

**Figure 5:**
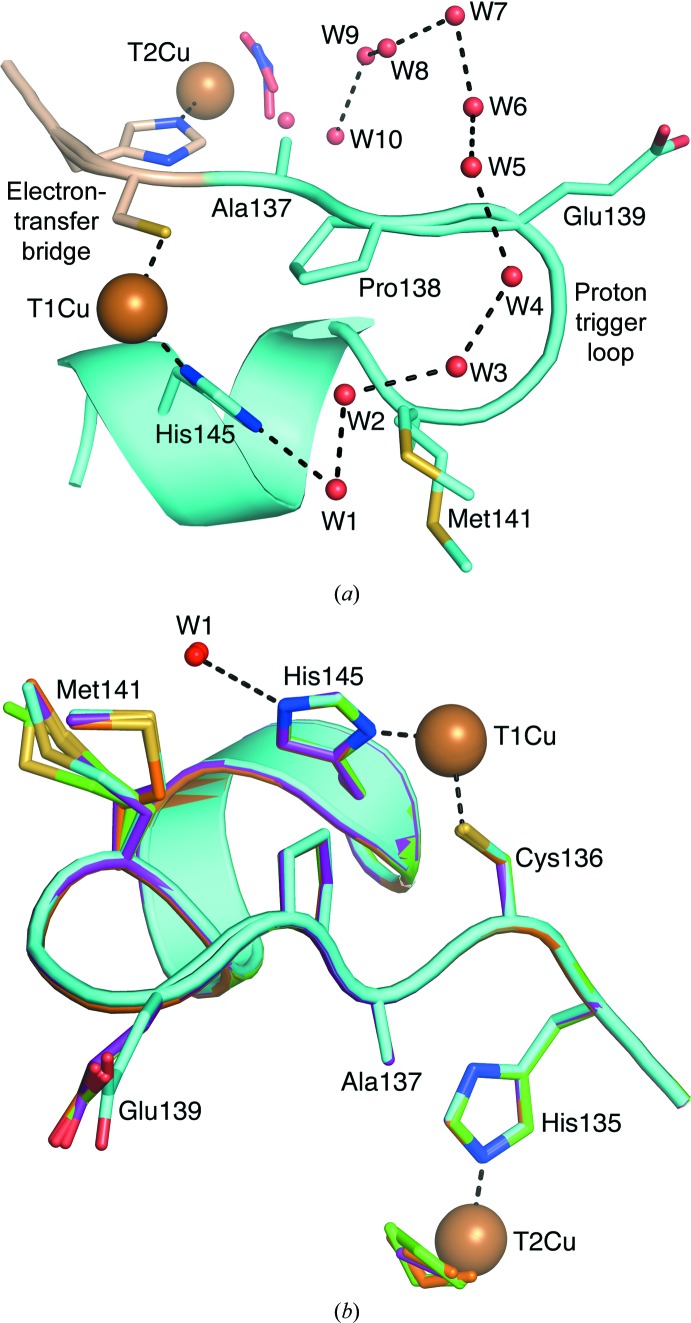
Coupling electron and proton transfer *via* the electron-transfer bridge and proton trigger loop. (*a*) Structure of *Ac*NiR ds11 from serial data collection showing the electron-transfer bridge (wheat), the proton trigger loop (cyan) and the water chain W1–W10 connecting the T1Cu and T2Cu sites to bulk solvent. Some partial-occupancy waters have been omitted for clarity. (*b*) Superposition of the electron-transfer bridge and proton trigger loop residues, showing multiple conformations of Met141 and the Glu139 side chains from serial data sets ds1 (green), ds11 (orange), ds17 (purple) and ds30 (cyan).

T1Cu–T2Cu electron transfer has been found to be coupled to proton transfer in the related blue *Ax*NiR (Leferink *et al.*, 2011 ▸) and the main proton channel was identified through an N90S mutation which reduced the enzymatic activity by ∼70% and caused significant structural rearrangements to reconstitute the hydrogen-bonding network. A well conserved water chain has been observed in our structural series consisting of up to ten water molecules spanning two copper sites and linking both sites to bulk solvent. This chain may act as a proton tube and access route to allow controlled delivery of protons to the T2Cu site in relation to the redox state of T1Cu. Several frames of a structural movie depicting changes to this water network from ds1 and ds40 are shown in Figs. 4 ▸(*a*)–4 ▸(*m*) and in the Supporting Information. Water W1 is located between Met141 and the T1Cu-liganding residue His145 and a complete water chain extending from W1 to W10 at T2Cu is observed in ds11 (Fig. 5 ▸ *a*), where the nitrite/nitric oxide/water ligand model was first observed (Fig. 1 ▸ *e*). Over the course of the series, Met141 shows several alternative conformations (Fig. 5 ▸ *b*). Met141 connects directly to the electron-transfer bridge (His135–Cys136) through a five-residue loop (Ala137–Met141), which we term the ‘proton trigger loop’. The hard-wired His–Cys electron-tranfer bridge shows a small (∼0.2 Å) shift in position between ds1 and ds30. This structural change appears to have propagated down the proton trigger loop, which shows a similar displacement between ds1 and ds30. Of particular note is the Glu139 side chain, which is highly mobile over the series, adopting several orientations (Fig. 5 ▸ *b*), but which shows no readily apparent correlation to ligand binding and turnover at the T2Cu. Future studies to investigate structural changes in this loop at temperatures of >100 K may provide further insight into how electron and proton transfer are coupled in CuNiRs.

## 4. Conclusions   

In this study, only one crystal diffracting to atomic resolution was used to probe the reaction mechanism of CuNiRs by exploiting the ability of X-ray-generated solvated electrons to drive the catalytic reaction. Use of the fast, shutterless Pilatus 6M-F detector allowed 45 serial crystallographic data sets to be measured in approximately 14 min within the Garman dose limit. Since the method can generate large volumes of data, automated data analysis is an essential requirement to avoid ‘data overload’. Ideally, automated analysis applies not only for an initial rapid assessment of data quality but for all steps needed to provide a ‘final’ refined and validated model. The automated data-analysis pipeline at Diamond Light Source has proved to be generally successful for rapid data reduction, although this work shows that some aspects remain challenging, such as inconsistent automated resolution cutoffs between successive data sets in the series. Inspection and re-processing of the automated output was necessary for some data sets used in this study, and in the end all data sets were re-processed using *XDS*. A challenge for automation of refinement during the model-building stage at each step in the series is to take into account the complex mix of radiation damage, gradual loss of resolution and changes in ligand identity through the enzymatic reaction. These challenges are likely to be addressed in the future, where developments in automated data handling and model building are being driven by high-throughput serial crystallography using XFELs and next-generation higher brightness synchrotron-radiation sources (*e.g.* ESRF Phase II and MAX IV; Borland, 2013 ▸; Eriksson *et al.*, 2014 ▸) and detectors. While XFELs have the potential for resolving part of this complex mix by yielding radiation-damage-free crystal structures (Chapman *et al.*, 2011 ▸), this objective needs to be achieved without also abolishing the photoelectrons needed for driving the chemical reaction, or alternative arrangements for inducing chemical change will be required. We anticipate that our approach could work well in parallel with rapid-mixing XFEL experiments.

We demonstrated that the accumulated X-ray dose drives the conversion of the substrate (nitrite) to the product (nitric oxide) *via* various intermediate stages prior to the loss of diffraction resolution owing to radiation damage. Variation in the T2Cu ligand density was observed over a number of data sets including two new conformations: ‘top hat’ and ‘side-on’ nitrite orientations pivoting about the central N atom and the dual-conformation nitrite/nitric oxide/water model observed in ds11 (38% of the Henderson limit). This technique has provided compelling evidence for the enzymatic generation of a stable side-on symmetrical binding conformation for T2Cu–nitric oxide, which is in contrast to proposals for an end-on binding mode based on a number of spectroscopic and computational studies (Periyasamy *et al.*, 2007 ▸; Sundararajan *et al.*, 2007 ▸; Silaghi-Dumitrescu, 2006 ▸; De Marothy *et al.*, 2007 ▸; Li *et al.*, 2015 ▸). Our data demonstrate that the side-on nitric oxide binding is generated enzymatically as proposed in Antonyuk *et al.* (2005 ▸). This binding mode is consistent with a DFT study that attributed it to steric interaction with Ile257 destabilizing the preferred end-on mode relative to the side-on structure (Merkle & Lehnert, 2009 ▸). Serial crystallographic structures have shown fluctuations in the occupancy of proximal and gatekeeper Asp98 with T2Cu ligands, suggesting a role in sensing, guiding or stabilizing the T2Cu substrate throughout the catalytic cycle. Changes have also been observed in the conserved water chain connecting the T1Cu and T2Cu sites to bulk solvent, which may act as a proton tube regulated by a proton trigger loop connected directly to the electron-transfer Cys–His bridge. Serial crystallography reveals conformational changes in Met141, the Cu-ligand residue at one end of the proton channel (tube), and Glu139, the function of which remains unclear. Overall, serial crystallography applied to X-ray-driven enzyme turnover is shown to be a powerful tool for defining structure-based enzyme mechanism *via* a continuous recording of structures as the redox reaction occurs. A major advantage of synchrotron-radiation serial crystallography is that it makes this approach feasible without the need for a large number of micro/nanocrystals or a large-sized single crystal.

## Supplementary Material

Additional details of data and modelling of structures.. DOI: 10.1107/S205225251600823X/lz5011sup1.pdf

Supplementary Movie S1.. DOI: 10.1107/S205225251600823X/lz5011sup2.mov

PDB reference: nitrite reductase, ds1, 5i6k

PDB reference: ds4, 5i6l

PDB reference: ds11, 5i6m

PDB reference: ds17, 5i6n

PDB reference: ds30, 5i6o

PDB reference: ds40, 5i6p

## Figures and Tables

**Table 1:** Crystallographic data-collection and structure-refinement statistics for selected serial data sets from the nitrite-soaked *Ac*NiR structural movie Values in parentheses are for the highest resolution shell. Data were processed using CC_1/2_ > 0.5 and an 〈*I*/σ(*I*)〉 > 1.0 (outer shell) cutoff.

Structure	ds1	ds4	ds11	ds17	ds30	ds40
Data collection						
Accumulated dose (MGy)	0.69	2.76	7.59	11.73	20.7	27.6
Unit-cell parameter (Å)	95.3	95.3	95.2	95.2	95.2	95.3
Resolution (Å)	42.6–1.07	42.6–1.08	42.6–1.09	42.6–1.22	42.6–1.45	42.6–1.56
Unique reflections†	121734 (5443)	119248 (5545)	115628 (5650)	82578 (4144)	48811 (2452)	39170 (2003)
Multiplicity	3.9 (2.7)	4.0 (3.0)	4.0 (3.3)	4.1 (3.8)	4.2 (4.2)	4.4 (4.2)
*R*_p.i.m._ (%)	2.6 (59.1)	2.4 (56.1)	2.2 (57.8)	2.1 (65.5)	2.3 (58.2)	2.6 (60.2)
CC_1/2_	0.54	0.50	0.57	0.53	0.55	0.52
〈*I*/σ(*I*)〉	13.4 (1.0)	14.8 (1.2)	15.9 (1.2)	17.5 (1.2)	16.1 (1.3)	14.3 (1.3)
Completeness (%)	96.7 (87.9)	97.4 (92.4)	97.5 (96.8)	97.4 (99.8)	96.2 (98.9)	95.7 (97.8)
Wilson *B* factor (Å^2^)	9.1	9.0	9.3	11.9	18.0	23.1
Refinement						
*R*_work_/*R*_free_ (%)	13.8/16.2	11.9/14.1	12.6/14.7	13.1/16.4	17.0/19.3	17.9/19.5
R.m.s.d., bond lengths (Å)	0.014	0.014	0.014	0.013	0.012	0.012
R.m.s.d., bond angles (°)	1.77	1.74	1.73	1.66	1.61	1.58
Maximum-likelihood-based ESU (Å)	0.02	0.02	0.02	0.03	0.05	0.06
Average protein *B* factor (Å^2^)	13.2	13.4	13.8	17.0	23.4	27.2
Average water *B* factor (Å^2^)	26.5	26.4	25.6	28.7	32.4	35.0
Ramachandran (%)						
Favoured regions	98.8	99.1	99.1	99.1	98.8	98.8
Allowed regions	1.2	0.9	0.9	0.9	1.2	1.2
PDB code	5i6k	5i6l	5i6m	5i6n	5i6o	5i6p

† Only partial reflections were recorded, *e.g.* for ds1 475 646 partials were recorded, while for ds40 164 925 partials were recorded.

**Table 2:** T2Cu site bond distances (Å) for selected serial data sets of nitrite-soaked *Ac*NiR

Structure	ds1	ds4	ds11	ds17	ds30	ds40
His100 N^∊2^	2.03	2.03	2.02	2.02	2.03	2.03
His135 N^∊2^	2.07	2.05	2.06	2.05	2.05	2.05
His306 N^∊2^	2.02	2.03	2.03	2.03	2.03	2.04
His255 N^∊2^	4.16	4.08	4.07	4.05	4.04	3.96
NO_2_a (O1/O2)	2.01/1.94	—	—	—	—	—
NO_2_b (O1/O2)	2.01/2.13	2.04/2.14	2.03/2.02	—	—	—
O/N (NO)	—	—	2.05/2.03	2.02/1.93	1.80/2.21	—
H_2_O	—	—	2.50	—	—	2.05
Asp98 OD2–NO_2_a (proximal)	2.38	2.26	—	—	—	—
Asp98 OD2–NO_2_b (proximal)	2.47	—	2.24	—	—	—
Asp98–NO (proximal)	—	—	2.34	2.65	2.84	—
Asp98 occupancy *A*/*B*	0.5/0.5	0.6/0.4	0.5/0.5	0.6/0.4	0.6/—	0.6/—
