# Supplementary material for: Serial crystallography captures enzyme catalysis in copper nitrite reductase at atomic resolution from one crystal
Source: IUCrJ. 2016 Jun 15;3(Pt 4):271–81. doi: 10.1107/S205225251600823X (PMC4937782; doi:10.1107/S205225251600823X)

IUCrJ

Volume 3 (2016)

Supporting information for article:

Serial crystallography captures enzyme catalysis in copper nitrite reductase at atomic resolution from one crystal

Sam Horrell, Svetlana V. Antonyuk, Robert R. Eady, S. Samar Hasnain, Michael A. Hough and Richard W. Strange

Table S1. T1Cu bond distances and angles for selected serial datasets of nitrite soaked *Ac*NiR

Structure	ds1	ds4	ds11	ds17	ds30	ds40
**Bond length (Å)**						
His95 Nδ1	2.05	2.04	2.04	2.04	2.05	2.05
His145 Nδ1	2.04	2.03	2.02	2.03	2.05	2.04
Cys136 Sγ	2.21	2.21	2.22	2.22	2.24	2.25
Met150 Sδ	2.52	2.49	2.49	2.47	2.43	2.39
**Bond angle (°)**						
His95 Nδ1-T1Cu-His145Nδ1	100	101	101	102	102	101
His145 Nδ1-T1Cu-Cys136 Sγ	106	108	107	108	108	109
Cys136 Sγ-T1Cu-Met150 Sδ	107	107	107	108	108	109
Met150 Sδ-T1Cu-His95 Nδ1	87	86	87	87	88	88
Cys136 Sγ-T1Cu-His95 Nδ1	131	129	129	128	127	128
Met150 Sδ-T1Cu-His145 Nδ1	129	127	127	125	124	122

Figure S1 Ligand binding at the T2Cu site. 4σ-weighted electron density Fo-Fc difference maps are shown for the datasets reported in Table 1 of the manuscript.

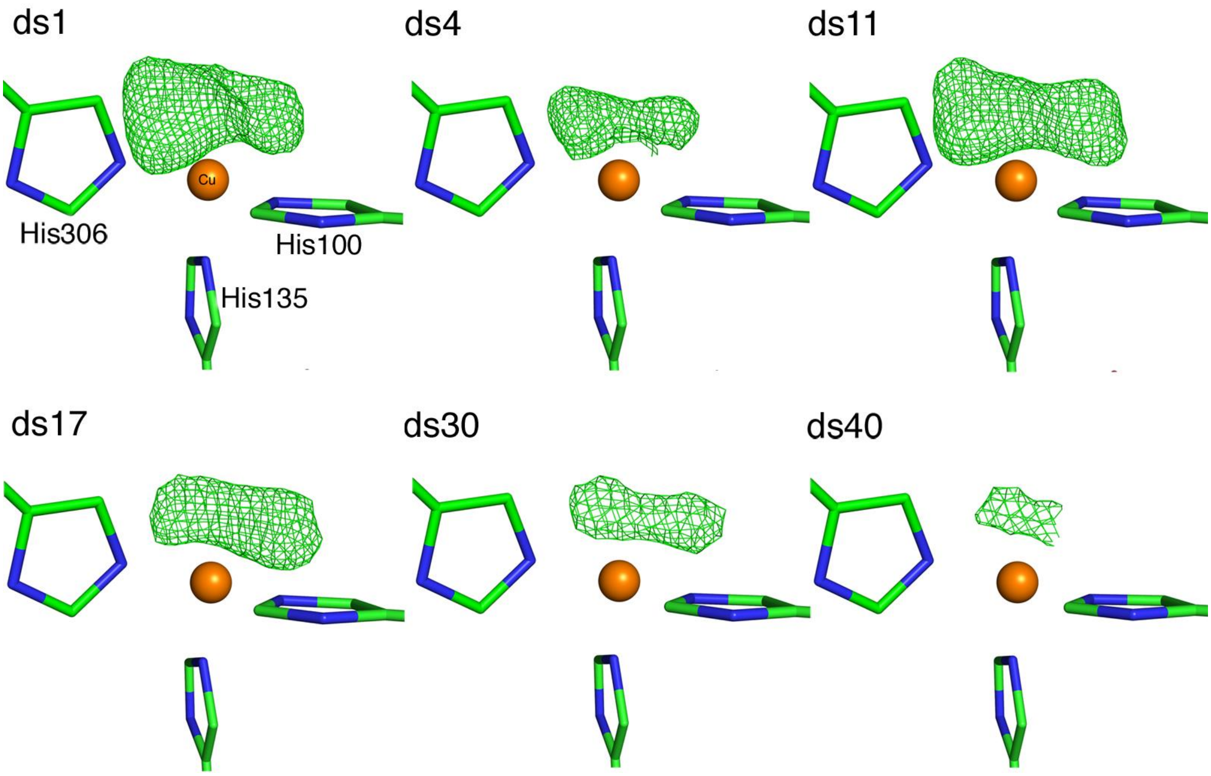

Figure S2 Ligand modelling for ds10 of the serial data collection with 2Fo-Fc density contoured to 1σ. All Asp98 residues are modelled at 40% and 60% for the gatekeeper and proximal conformations, respectively. a) A single side-on nitrite molecule at 70% occupancy. b) A single side-on nitrite molecule at 70% occupancy with waters W1 and W2 modelled at 20% and 80% occupancy, respectively. c) Dual occupancy nitrite and nitric oxide molecules at 33% each with waters W1 and W2 at 33% and 80% occupancy, respectively. d) Dual occupancy nitrite and nitric oxide at 33% occupancy each with nitric oxide in an end on orientation and a water molecule at 80% occupancy. e) Dual occupancy nitrite and nitric oxide molecules at 33% occupancy with a second nitric oxide molecule in the end on conformation at 33% occupancy. f) Dual occupancy nitric oxide molecules at 35% occupancy with one in an end on conformation. d) Dual occupancy nitric oxide molecules at 35% occupancy with one in an end on conformation with a water molecule at 80% occupancy. Asp98, nitrite and nitric oxide are represented as sticks and water molecules and copper atoms as spheres. All atoms are coloured by element.

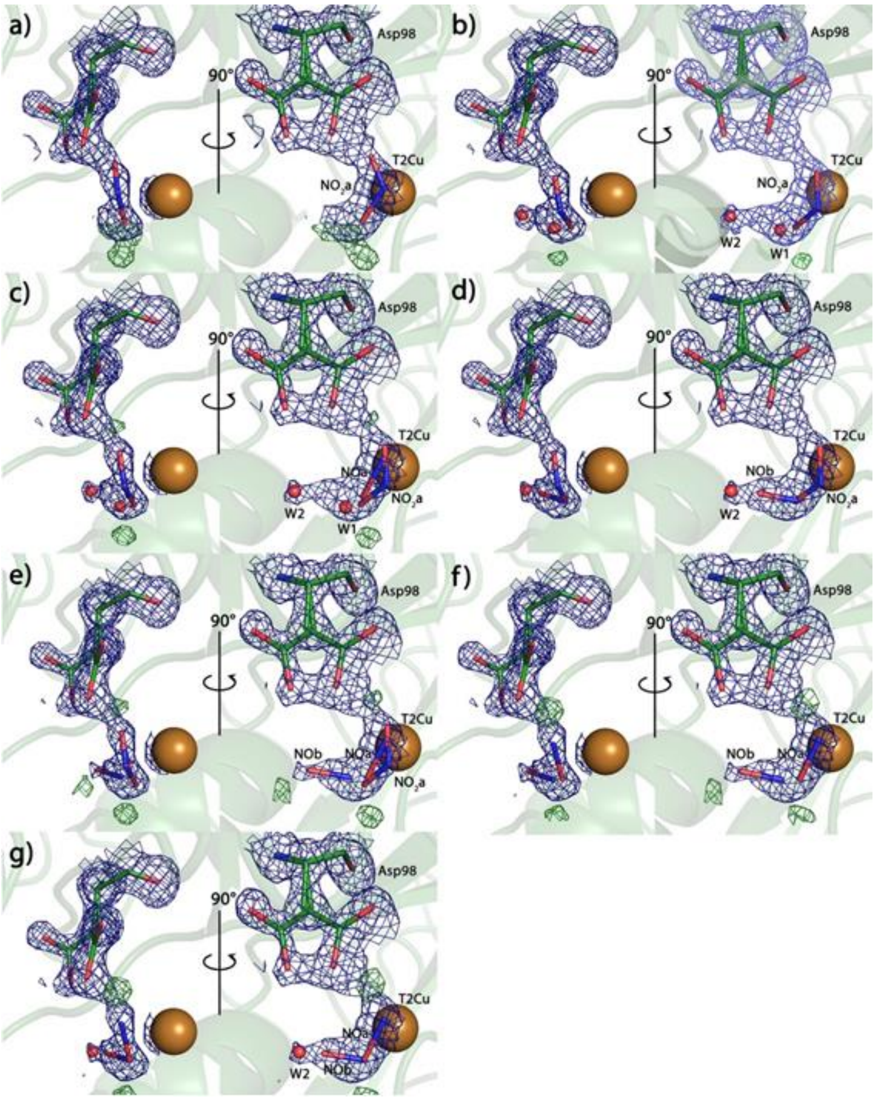
a)
Asp98
90°
T2Cu
NO_2a
b)
Asp98
90°
T2Cu
NO_2a
W2
W1
c)
Asp98
90°
T2Cu
W2
W1
NO_2a
d)
Asp98
90°
T2Cu
NOb
W2
NO_2a
e)
Asp98
90°
T2Cu
NOb
NO_2a
f)
Asp98
90°
T2Cu
NOb
g)
Asp98
90°
T2Cu
W2
NOb

Figure S3. Ligand and Asp98 conformation occupancy plotted against dataset number. a) T2Cu ligand occupancy with total nitrite occupancy (the sum of multiple conformations when present) as green triangles, nitric oxide occupancy as orange circles and water occupancy as purple diamonds. b) Asp98 alternate conformation occupancies with the proximal conformation as red squares and the gatekeeper conformation as blue diamonds.

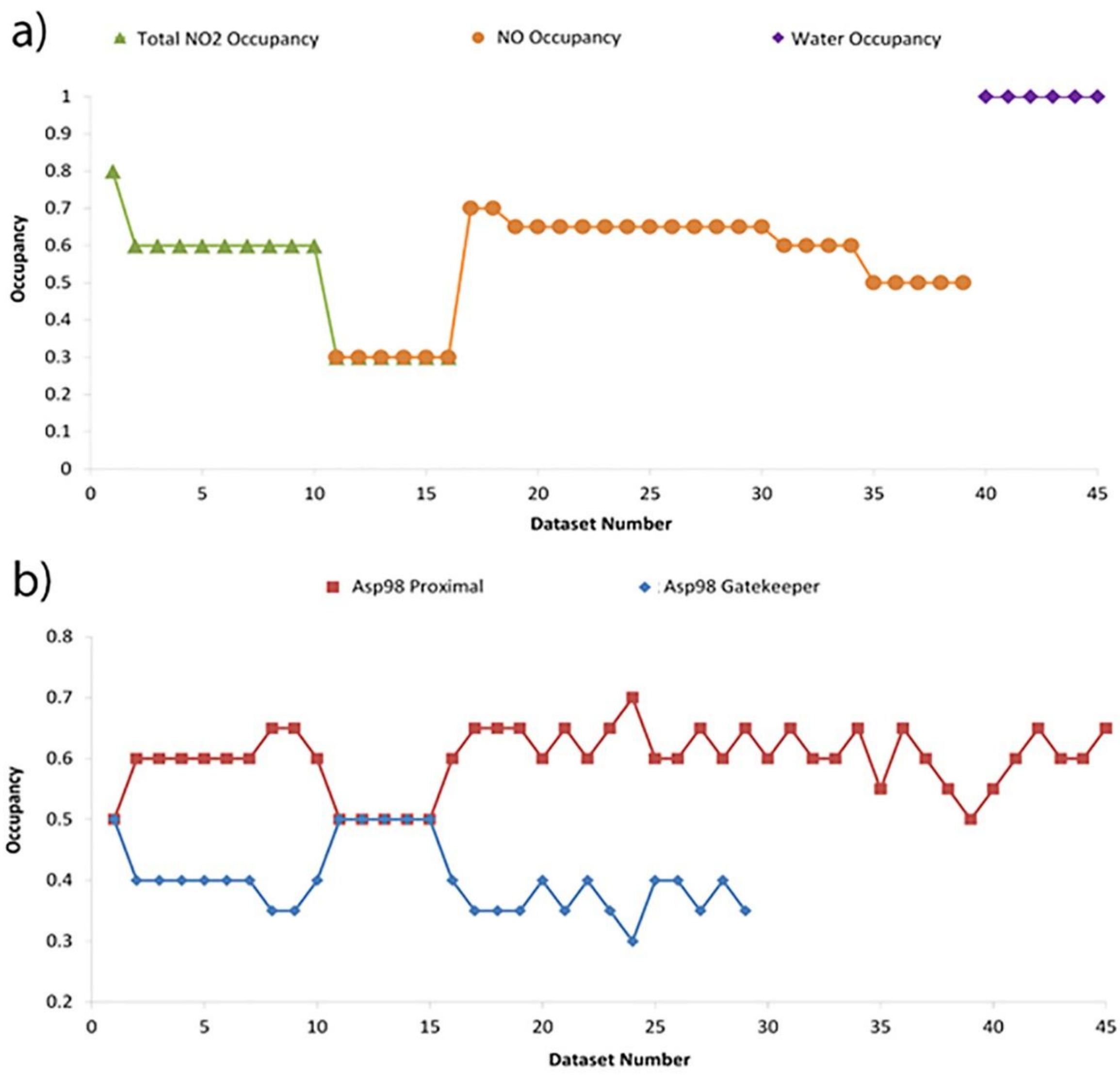

Figure S4. Superposition of the T2Cu copper site of datasets from serial data collection with nitrite bound ds1 (green), side on nitrite bound ds4 (wheat), nitrite and nitric oxide bound ds11 (orange), nitric oxide bound ds17 (purple) and nitric oxide bound ds30 (cyan). Residues and ligands are represented as sticks and copper atoms as spheres.

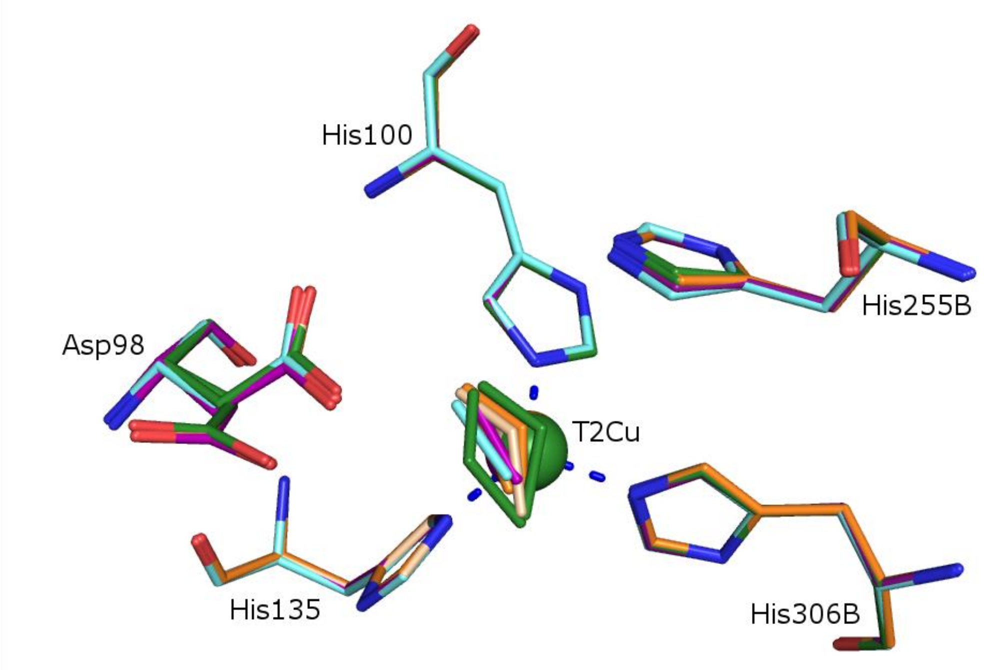

Supplement: Supplementary file 1 [file m-03-00271-sup1.pdf]
